# Patient‐Perceived Benefits of Named‐Patient Product Sublingual Immunotherapy in Allergic Rhinitis and Asthma: Primary Results From the ERAPP Real‐World Cohort Study

**DOI:** 10.1111/all.70270

**Published:** 2026-03-08

**Authors:** Davide Caimmi, Abdelilah Abouelfath, Régis Lassalle, Séverine Lignot‐Maleyran, Emmanuelle Bignon, Patrick Blin, Evangéline Clark, Julien Cottet, Laure Carcaillon‐Bentata, Pascal Demoly

**Affiliations:** ^1^ Allergy Unit, Hôpital Arnaud De Villeneuve University Hospital of Montpellier Montpellier Occitanie France; ^2^ IDESP, UMR 1318 University of Montpellier – INSERM Montpellier Occitanie France; ^3^ Bordeaux Pharmaco Epi, INSERM CIC‐P 1401 Université De Bordeaux Bordeaux France; ^4^ Allergy Unit Hospital of Mont De Marsan Mont‐de‐Marsan Nouvelle‐Aquitaine France; ^5^ Private Allergy Practice Chartres France; ^6^ INRIA PreMediCal Montpellier France

**Keywords:** NPP, patient's benefit, PROMs, questionnaires, sublingual allergen immunotherapy

## Abstract

**Background:**

Named‐patient product sublingual immunotherapy (NPP‐SLIT) is widely used in France, yet real‐world evidence on patient‐perceived benefit remains limited.

**Objective:**

To assess treatment expectations and patient‐perceived benefit over 12–15 months among recent NPP‐SLIT initiators using the Patient Benefit Index (PBI) and validated patient‐reported outcome measures (PROMs).

**Methods:**

ERAPP is a prospective, multicenter, observational study in children and adults with IgE‐mediated respiratory allergy. Initiators (≤ 6 months on NPP‐SLIT at baseline) completed digital PROMs at baseline, Month 6, and Month 12–15. The primary endpoint was the proportion with PBI ≥ 1 at Months 12–15. Secondary endpoints were changes in PROMs; exploratory analyses examined higher PBI thresholds and item‐level fulfillment.

**Results:**

Of 9439 enrolled, 4794 were initiators (950 children; 3844 adolescents/adults). At Month 12–15, PBI ≥ 1 was achieved by 83.8% of children and 84.0% of adolescents/adults. Symptom burden (T5SS) and rhinitis severity (ARIA) improved beyond published MIDs. Asthma control (ACT) improved, whereas changes in rhinitis control (ARCT, ≥ 12 years) and daytime sleepiness (ESS, adults) were below their respective MIDs. Treatment satisfaction (ESPIA‐Q11) increased, while adherence (GIRERD) decreased from baseline to follow‐up. Nearly half reached PBI ≥ 2 and about one‐fifth PBI ≥ 3. Item‐level analyses showed highest fulfillment for nasal obstruction relief, improved sleep, and overall symptom relief; fatigue, mood, and social aspects were less frequently fulfilled. Children generally reported slightly higher fulfillment than adolescents/adults.

**Conclusion:**

NPP‐SLIT provides sustained, clinically meaningful benefit from the patient's perspective in both age groups. ERAPP supports the PBI as a complementary endpoint that links patient expectations to outcomes and informs patient‐centered allergy care.

## Graphical abstract

Evaluation en vie Réelle de l'Allergo‐Prescription en Pratique (ERAPP) is a large, prospective, real‐world cohort of children and adults initiating named‐patient product sublingual immunotherapy (NPP‐SLIT) in France. More than 80% of patients reported a clinically meaningful, expectation‐anchored benefit (Patient Benefit Index ≥ 1) at 12–15 months. The Patient Benefit Index complements conventional patient‐reported outcome measures by capturing what matters most to patients in routine allergy care. ACT, asthma control test; ARIA, allergic rhinitis and its impact on asthma; SLIT, sublingual immunotherapy; T5SS, total 5 symptom score.

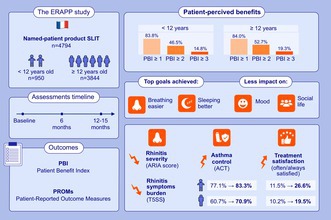

**Abbreviations**
ACT: Asthma Control Test
AIT: Allergen Immunotherapy
ARCT: Allergic Rhinitis Control Test
ARIA: Allergic Rhinitis and its Impact on Asthma
ERAPP: Real‐world study on patient‐perceived outcomes of Named‐Patient Products (originally: Étude sur le Ressenti des APSI Par le Patient)
ESPIA: Satisfaction Scale for Patients Undergoing Allergen Immunotherapy (originally developed in Spanish and translated into French: Échelle de Satisfaction des Patients vis‐à‐vis de leur Immunothérapie Allergénique)
ESS: Epworth Sleepiness Scale
GIRERD: Adherence Questionnaire by Girerd
HTA: Health Technology Assessment
NPP‐SLIT: Pre‐packed liquid allergen extracts prepared for individual prescriptions
PBI: Patient Benefit Index
PBQ: Patient Benefit Questionnaire
PNQ: Patient Need Questionnaire
PROMs: Patient‐Reported Outcome Measures
PROs: Patient‐Reported Outcomes
RCTs: Randomized Clinical Trials
SLIT: Sublingual Immunotherapy
SNDS: Système National des Données de Santé (France)
T5SS: Total 5 Symptom Score

## 1 Introduction

Allergic rhinitis and asthma are among the most prevalent chronic respiratory diseases, affecting patients of all ages and substantially impairing daily functioning. Allergen immunotherapy (AIT) is an etiological intervention for allergic rhinitis and asthma, aiming not only to relieve symptoms but also to modulate the immune response and alter disease progression [1, 2]. Among the available modalities, sublingual immunotherapy (SLIT) is widely prescribed in Europe due to its favorable safety profile and patient convenience. In France, a commonly used form of SLIT is delivered as a named‐patient product (NPP‐SLIT), a liquid formulation prescribed on an individual basis and prepared by authorized companies. In this context, named‐patient products (also referred to as allergen extracts prepared for an individual patient; Allergènes Préparés Spécialement pour un Individu, APSI) are prepared for an individual patient on prescription within a dedicated national framework.

While its clinical use is widespread, real‐world evidence on patient‐perceived effectiveness remains limited. Controlled trials and real‐life studies have demonstrated the clinical efficacy and safety of SLIT in children and adults [3, 4, 5], and their controlled settings and strict eligibility criteria may not fully reflect routine practice. In this context, patient‐reported outcome measures (PROMs) and instruments such as the Patient Benefit Index (PBI) provide a complementary, patient‐centered evaluation of treatment value.

Despite strong evidence for SLIT efficacy from randomized trials, current evaluation frameworks often rely on symptom scores or physician‐reported endpoints, which may not fully capture individual patient goals or perceptions. In real‐world settings, patients may prioritize aspects such as daily functioning, sleep quality, or ease of use over classical symptom‐based endpoints. Thus, there is a need for more comprehensive outcome measures that reflect real‐life impact and value.

The PBI is a validated tool that quantifies the extent to which patients perceive that their personal treatment goals have been achieved [6]. Unlike standard PROMs, the PBI links baseline expectations with reported benefit, offering a patient‐defined measure of therapeutic value. It has been successfully applied in allergy studies and complements conventional clinical endpoints by capturing what matters most to patients in previous observational research [7].

The ERAPP study (*Évaluation en vie Réelle de l'Allergo‐Prescription en Pédiatrie et en Pratique*.) was designed to assess real‐world patient benefit, disease control, sleep and daily functioning, treatment adherence, and NPP‐SLIT treatment satisfaction. Using a comprehensive set of validated PROMs, the ERAPP study followed individuals treated with NPP‐SLIT over a 12‐ to 15‐month period, in both pediatric and adult populations. The objective of the present analysis is to evaluate treatment expectations and patient‐perceived benefit of NPP‐SLIT over 12–15 months, with the aim to inform clinical practice, supporting individualized care, and providing evidence relevant to healthcare decision‐makers, especially in terms of treatment value in routine practice.

## 2 Methods

The design and rationale of the ERAPP study have been described previously [8]. ERAPP is a prospective, multicenter, observational cohort conducted in routine allergy practices across France. Eligible patients had a confirmed diagnosis of IgE‐mediated respiratory allergy (rhinitis, conjunctivitis, and/or asthma) and received a prescription for prepacked liquid NPP‐SLIT between September 2020 and February 2022. Prescriptions could include one or more allergen extracts, as part of routine clinical practice. The present analysis focused exclusively on initiators, defined as patients who had been on NPP‐SLIT for ≤ 6 months at baseline. Patients already treated for > 6 months (*n* = 4645) were excluded from this analysis.

Data were collected electronically via a secure digital platform at baseline (T0), Month 6 (T6), and Month 12–15 (T12–15). Participants (or caregivers, for children) completed a comprehensive set of validated PROMs, including

- the Patient Needs Questionnaire (PNQ) and the Patient Benefit Questionnaire (PBQ), used to derive the PBI [6, 7], as per protocol, the PNQ was administered only to initiators, making PBI calculation applicable solely in this subgroup.
- the Allergic Rhinitis and its Impact on Asthma (ARIA) score for rhinitis severity [9];
- the Asthma Control Test (ACT) [10];
- the Allergic Rhinitis Control Test (ARCT), a validated five‐item tool for patients aged ≥ 12 years [11]; the same version was used for adolescents and adults. A pediatric adaptation including three additional items, under development, was not analyzed in this study—Study ongoing (RCB # 2016‐A01504‐47, EC approval 10–04‐17);
- the Total 5 Rhinitis Symptom Score (T5SS) [12];
- the 11th question on quality of life of the Satisfaction with Allergen Immunotherapy (ESPIA) scale [13];
- the Epworth Sleepiness Scale (ESS) [14];
- and the GIRERD adherence questionnaire [15].

All questionnaires were completed independently online, under standardized conditions. To support the interpretation of PROM changes, literature‐based minimal important difference (MID) thresholds are summarized in Supplementary Table S1. These serve as interpretive references, not predefined analytical cutoffs.

The primary outcome was the proportion of patients achieving a PBI score ≥ 1 at 12–15 months, reflecting clinically meaningful benefit. Analyses included individuals having both 12 to 15 months PBQ and baseline PNQ available questionnaires. As PNQ was administered only to initiators, PBI was evaluable solely in this subgroup. Secondary outcomes included changes of PROM scores over time (ARIA, ACT, ARCT, T5SS, ESS, ESPIA‐Q11, and GIRERD).

Additional exploratory analyses were conducted to provide further descriptive insights. These included the following:

- proportion of patients achieving a PBI score ≥ 1 at 6 months, reflecting clinically meaningful benefit.
- higher thresholds of perceived benefit (PBI ≥ 2 and PBI ≥ 3) at 12–15 months.
- item‐level analyses of PNQ/PBQ expectations at baseline and their fulfillment at 6 and 12–15 months.
- and subgroup analyses of PBI ≥ 1 at 12–15 months according to demographic and clinical categories (sex, asthma comorbidity, baseline ARIA, T5SS, and ACT classes)

These exploratory analyses were based on complementary field analyses of the PNQ/PBQ data and were not prespecified in the SAP.

All analyses were descriptive. Categorical variables are presented as counts and percentages and continuous variables as means with standard deviations. Missing data were not imputed. PROM scores were summarized at each timepoint, with changes described in absolute terms. No formal hypothesis testing was prespecified.

## 3 Results

### 3.1 Study Population

A total of 9439 patients were included in the ERAPP study. Of these, 4794 were classified as NPP‐SLIT initiators (treatment duration ≤ 6 months at inclusion) and constituted the primary analysis population. Among them, 950 (19.8%) were children (< 12 years) and 3844 (80.2%) were adolescents or adults (≥ 12 years). The baseline demographic and clinical characteristics of NPP‐SLIT initiators, including age, sex, residence, smoking status, allergy history, comorbid asthma, indication for treatment, and attrition across time‐points, are summarized in Table 1. Prescribed allergen groups were predominantly house dust mite and grass/tree pollens; multiple allergens could be prescribed per patient (Table 1).

**TABLE 1:** Characteristics of the study population (NPP‐SLIT initiators only) and attrition across time‐points, stratified by age group (< 12 vs. ≥ 12 years).

Characteristics	Children < 12y (*n* = 950)	Adolescents/Adults ≥ 12y (*n* = 3844)
Female sex, *n* (%)	325 (34.2)	2327 (60.5)
Mean age, years (±SD)	8.4 (1.9)	34.2 (14.6)
Urban residence, *n* (%)	473 (49.8)	2412 (62.7)
Non‐smoker, *n* (%)	935 (98.4)	2956 (76.9)
History of allergy > 10y, *n* (%)	8 (0.8)	2134 (55.7)
Asthma as comorbidity, *n* (%)	258 (27.2)	756 (19.7)
AIT prescribed for asthma, *n* (%)	464 (48.8)	1332 (34.7)
Main indication: allergic rhinitis, *n* (%)	786 (82.7)	3469 (90.2)
Polysensitized, *n* (%)	202 (21.3)	1208 (31.4)
Prescribed allergen: house dust mite, *n* (%)	583 (61.4)	1369 (35.6)
Prescribed allergen: grass pollen, *n* (%)	320 (33.7)	1740 (45.3)
Prescribed allergen: tree pollen, *n* (%)	196 (20.6)	1426 (37.1)
Prescribed allergen: other(s), *n* (%)	109 (11.5)	850 (22.1)
Evaluable PNQ, *n* (%)	933 (98.2)	3783 (98.4)
Evaluable PBI at Month 6, *n* (%)	532 (56.0)	1942 (50.5)
Evaluable PBI at Month 12–15, *n* (%)	439 (46.2)	1371 (35.7)

Abbreviations: PNQ: Patient Needs Questionnaire; PBQ: Patient Benefit Questionnaire; PBI: Patient Benefit Index. For each questionnaire (PNQ and PBQ), both the number of collected and evaluable responses are reported. Evaluable responses were defined as having no missing items or a maximum of 6 missing responses. Allergen categories are not mutually exclusive; multiple allergens could be prescribed per patient.

### 3.2 Primary Outcome—Patient‐Perceived Benefit

**FIGURE 1:**
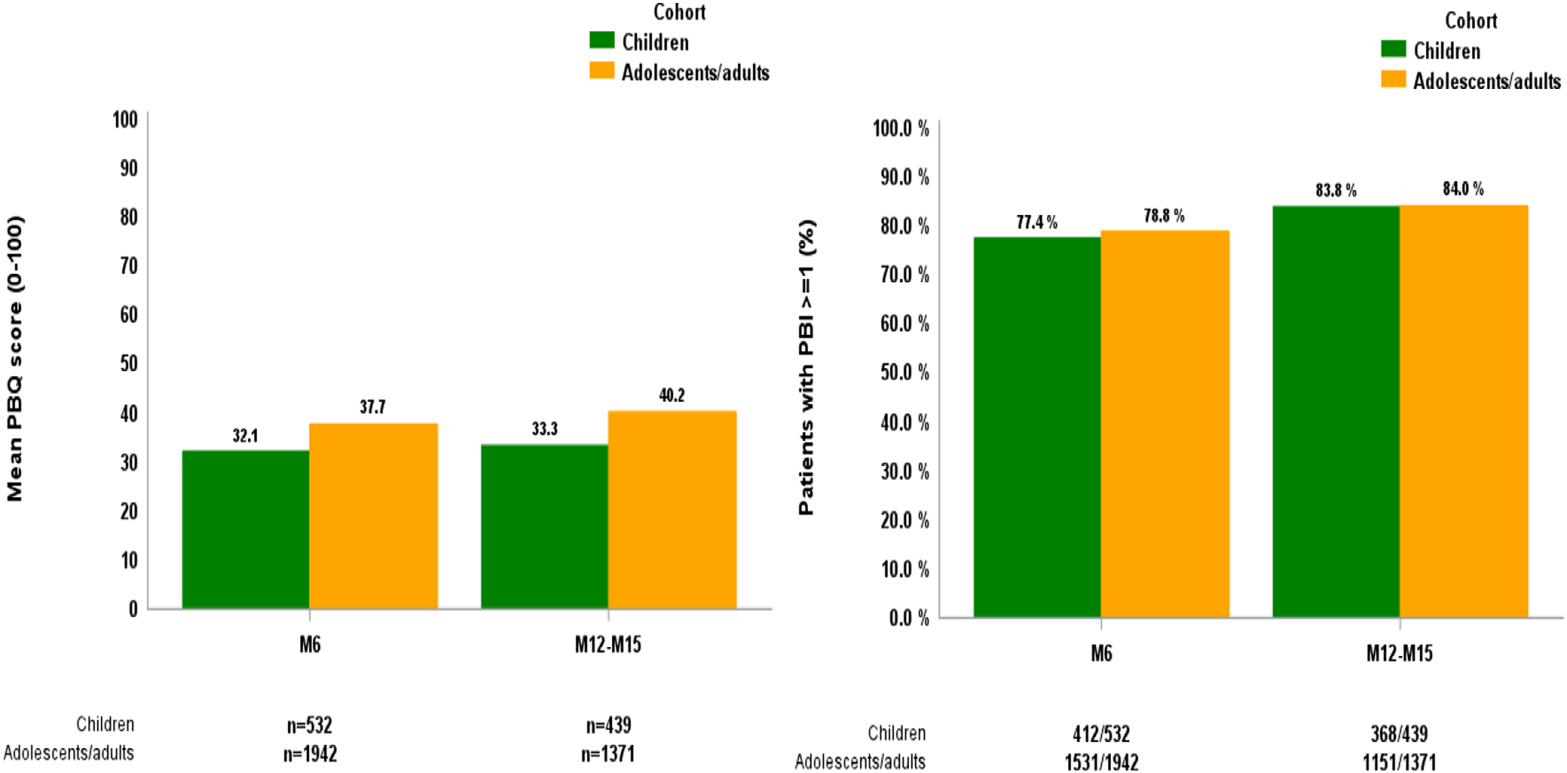
Evolution of PBQ scores and patient‐perceived benefit (PBI) over time among NPP‐SLIT initiators (≤ 6 months at baseline) with evaluable PNQ/PBQ data, by age group. Bar charts present the mean symptom burden scores (PBQ; left side) and the proportion of patients achieving a clinically meaningful benefit (PBI ≥ 1; right side) at Month 6 and Month 12–15 among children (< 12 years) and adolescents/adults (≥ 12 years) initiating NPP‐SLIT. Analyses included only initiators (≤ 6 months at baseline) with evaluable PNQ/PBQ.

The primary outcome was the proportion of initiators achieving a clinically meaningful benefit, defined as a PBI ≥ 1 at Months 12–15. This threshold was reached by 83.8% (368/439) of children and 84.0% (1151/1371) of adolescents/adults (Figure 1). At Month 6, the corresponding proportions were 77.4% (412/532) and 78.8% (1531/1942), confirming a sustained and consistently high level of perceived benefit over time.

### 3.3 Secondary Outcomes—Symptom Trajectories and PROMs

Changes in PROMs are summarized in Table 2. PROMs improved from baseline to follow‐up, particularly for rhinitis severity and symptom burden. ARIA decreased from 2.5 ± 1.2 (*n* = 828 a) at baseline to 1.9 ± 1.3 (*n* = 343 a) at Months 12–15 in children and from 2.6 ± 1.1 (*n* = 3559 a) to 2.1 ± 1.3 (*n* = 1165 a) in adolescents/adults. T5SS decreased from 11.3 ± 3.8 (*n* = 827 a) to 8.1 ± 4.2 (*n* = 342 a) in children and from 12.7 ± 3.6 (*n* = 3546 a) to 9.2 ± 4.3 (*n* = 1161 a) in adolescents/adults. Among patients with asthma and evaluable ACT, the proportion with controlled asthma increased from 77.1% at baseline to 83.3% at Month 12–15 in children and from 60.7% to 70.9% in adolescents/adults. ARCT increased modestly from 18.3 ± 4.6 (*n* = 3561 a) to 20.3 ± 4.0 (*n* = 1167 a) (≥ 12 years), and ESS decreased from 8.5 ± 4.9 (*n* = 3160 c) to 7.4 ± 4.5 (*n* = 1180 c) (adults). Treatment satisfaction (ESPIA‐Q11 “often/always”) increased from 11.5% (*n* = 109 d) to 26.6% (*n* = 253 d) in children and from 10.2% (*n* = 393 d) to 19.5% (*n* = 750 d) in adolescents/adults, whereas adherence (GIRERD) declined over time (children: observant 34.8% at baseline vs. 11.2% at Month 12–15; adolescents/adults: 42.1% vs. 19.6%).

**TABLE 2:** Summary of patient‐reported outcome measures (PROMs) and disease control in NPP‐SLIT initiators.

PROM/Tool	Population	Baseline	Month 6	Month 12–15	Interpretation
ARIA (rhinitis severity, 0–4)	Children < 12y	2.5 ± 1.2 (*n* = 828^a^)	2.0 ± 1.3 (*n* = 431^a^)	1.9 ± 1.3 (*n* = 343^a^)	Consistent reduction in symptom severity
	Adolescents/Adults ≥ 12y	2.6 ± 1.1 (*n* = 3559^a^)	2.2 ± 1.3 (*n* = 1708^a^)	2.1 ± 1.3 (*n* = 1165^a^)	
ACT (categorical distribution of asthma control, % of patients^b^)	Children < 12y	Controlled 77.1%; Partly controlled 18.8%; Uncontrolled 4.1%	Controlled 81.6%; Partly controlled 17.3%; Uncontrolled 1.2%	Controlled 83.3%; Partly controlled 13.7%; Uncontrolled 3.1%	Improved asthma control over time (higher proportion controlled)
	Adolescents/Adults ≥ 12y	Controlled 60.7%; Partly controlled 26.5%; Uncontrolled 12.8%	Controlled 67.9%; Partly controlled 25.8%; Uncontrolled 6.3%	Controlled 70.9%; Partly controlled 22.4%; Uncontrolled 6.7%	
ARCT (rhinitis control, 5–25)	Adolescents/Adults ≥ 12y	18.3 ± 4.6 (*n* = 3561^a^)	19.2 ± 4.1 (*n* = 1711^a^)	20.3 ± 4.0 (*n* = 1167^a^)	Modest improvement below MID
T5SS (total rhinitis symptom score, 0–20)	Children < 12y	11.3 ± 3.8 (*n* = 827^a^)	8.3 ± 3.9 (*n* = 427^a^)	8.1 ± 4.2 (*n* = 342^a^)	Reduction > MID of 2 points
	Adults ≥ 12y	12.7 ± 3.6 (*n* = 3546^a^)	9.4 ± 4.2 (*n* = 1703^a^)	9.2 ± 4.3 (*n* = 1161^a^)	
ESS (Epworth Sleepiness Scale, 0–24)	Adults ≥ 18y	8.5 ± 4.9 (*n* = 3160^c^)	7.9 ± 4.7 (*n* = 1648^c^)	7.4 ± 4.5 (*n* = 1180^c^)	Modest improvement below MID
ESPIA‐Q11 (quality of life impact, % “often/always”)	Children < 12y	11.5% (*n* = 109^d^)	26.7% (*n* = 254^d^)	26.6% (*n* = 253^d^)	Increased satisfaction over time
	Adolescents/Adults ≥ 12y	10.2% (*n* = 393^d^)	23.2% (*n* = 891^d^)	19.5% (*n* = 750^d^)	Increased satisfaction over time
GIRERD (adherence to NPP‐SLIT, % of patients^e^)	Children < 12y	Observant 34.8%; Partially observant 57.6%; Non‐observant 7.7%	Observant 12.9%; Partially observant 67.5%; Non‐observant 19.5%	Observant 11.2%; Partially observant 65.1%; Non‐observant 23.7%	Decrease in adherence between baseline and both follow‐up visits
	Adolescents/Adults ≥ 12y	Observant 42.1%; Partially observant 50.5%; Non‐observant 7.4%	Observant 18.6%; Partially observant 66.1%; Non‐observant 15.3%	Observant 19.6%; Partially observant 63.6%; Non‐observant 16.8%	

*Note:* Values are presented as mean ± SD unless otherwise specified. For ESPIA‐Q11 (treatment satisfaction, quality‐of‐life item) and GIRERD (adherence), data are expressed as proportions. MID: minimal, important difference. Evaluable: all responses to the items in the questionnaire (ARIA, T5SS, ARCT, ACT, GIRERD or ESS) are completed.

^a^ Number of patients with allergic rhinitis and the evaluable concerned questionnaire (ARIA, T5SS or ARCT) at the measure points: baseline or baseline and 6 months or baseline and 12–15 months.

^b^ % Among patients with asthma and the evaluable ACT questionnaire at the measure points: baseline or baseline and 6 months or baseline and 12–15 months.

^**c**^ Number of adults **≥** 18y with evaluable Epworth questionnaire at the measure points; baseline or baseline and 6 months or baseline and 12–15 months.

^d^ Number of patients with ESPIA questionnaire at the measure points; baseline or baseline and 6 months or baseline and 12–15 months.

^**e**^ % Among patients with evaluable **GIRERD** questionnaire at the measure points: baseline or baseline and 6 months or baseline and 12–15 months.

**TABLE 3:** Proportion of NPP‐SLIT initiators achieving a clinically meaningful benefit (PBI ≥ 1) at Month 6 and Months 12–15, stratified by demographic and clinical subgroups (exploratory analyses).

Subgroup	Children < 12y n/N, (%)		Adolescents/Adults ≥ 12y n/N, (%)	
	Month 6	Month 12–15	Month 6	Month 12–15
**Sex**				
Male	266/345 (77.1%)	241/286 (84.3%)	632/786 (80.4%)	477/557 (85.6%)
Female	146/186 (78.5%)	127/152 (83.6%)	899/1155 (77.8%)	674/814 (82.8%)
**Asthma/Rhinitis‐Conj**.				
Asthma – Yes	223/296 (75.3%)	212/259 (81.9%)	677/848 (79.8%)	510/610 (83.6%)
Rhinitis/Conj. – Yes	363/467 (77.7%)	325/388 (83.8%)	1446/1832 (78.9%)	1090/1293 (84.3%)
**ARIA at baseline**				
ARIA = 0	4/10 (40.0%)	9/14 (64.3%)	21/29 (72.4%)	18/21 (85.7%)
ARIA = 1	56/79 (70.9%)	51/64 (79.7%)	194/260 (74.6%)	160/194 (82.5%)
ARIA = 2	136/180 (75.6%)	177/146 (80.1%)	453/588 (77.0%)	365/437 (83.5%)
ARIA = 3	70/88 (79.5%)	72/80 (90.0%)	355/438 (81.1%)	262/307 (85.3%)
ARIA = 4	92/107 (86.0%)	72/82 (88.9%)	417/512 (81.4%)	279/332 (84.0%)
**T5SS at baseline**				
0–7	53/78 (67.9%)	61/69 (88.4%)	122/162 (75.3%)	100/125 (80.0%)
8–11	97/129 (75.2%)	96/115 (83.5%)	266/355 (74.9%)	196/254 (77.2%)
11–13	78/93 (83.9%)	63/72 (87.5%)	284/354 (80.2%)	215/249 (86.3%)
13–20	96/116 (82.8%)	73/91 (80.2%)	586/726 (80.7%)	439/508 (86.4%)
**ARCT at baseline (≥ 12 y)**				
Rhinitis uncontrolled	_	_	808/1023 (79.0%)	624/741 (84.2%)
Rhinitis controlled	_	_	632/805 (78.5%)	459/549 (83.6%)
**ACT at baseline (asthma)**				
Uncontrolled	13/13 (100.0%)	9/11 (81.8%)	78/100 (78.0%)	58/74 (78.4%)
Partly controlled	45/62 (72.6%)	41/50 (82.0%)	163/208 (78.4%)	132/158 (83.5%)
Controlled	164/220 (74.5%)	161/197 (81.7%)	433/537 (80.6%)	318/376 (84.6%)

*Note:* Data are presented as n/N (%) among initiators (≤ 6 months of treatment at baseline) with at least two evaluable PNQ/PBQ questionnaires (baseline and one follow‐up). Subgroups include sex, asthma comorbidity, baseline rhinitis severity (ARIA), baseline symptom burden (T5SS), baseline asthma control (ACT), and rhinitis control (ARCT, ≥ 12 years only). These analyses were descriptive, based on complementary PNQ/PBQ analyses, and were not prespecified in the SAP. Percentages are calculated as n/*N* × 100 and rounded to one decimal place. PBI: Patient Benefit Index; PNQ: Patient Needs Questionnaire; PBQ: Patient Benefit Questionnaire; PROM: patient‐reported outcome measure.

### 3.4 Exploratory Analysis

**FIGURE 2:**
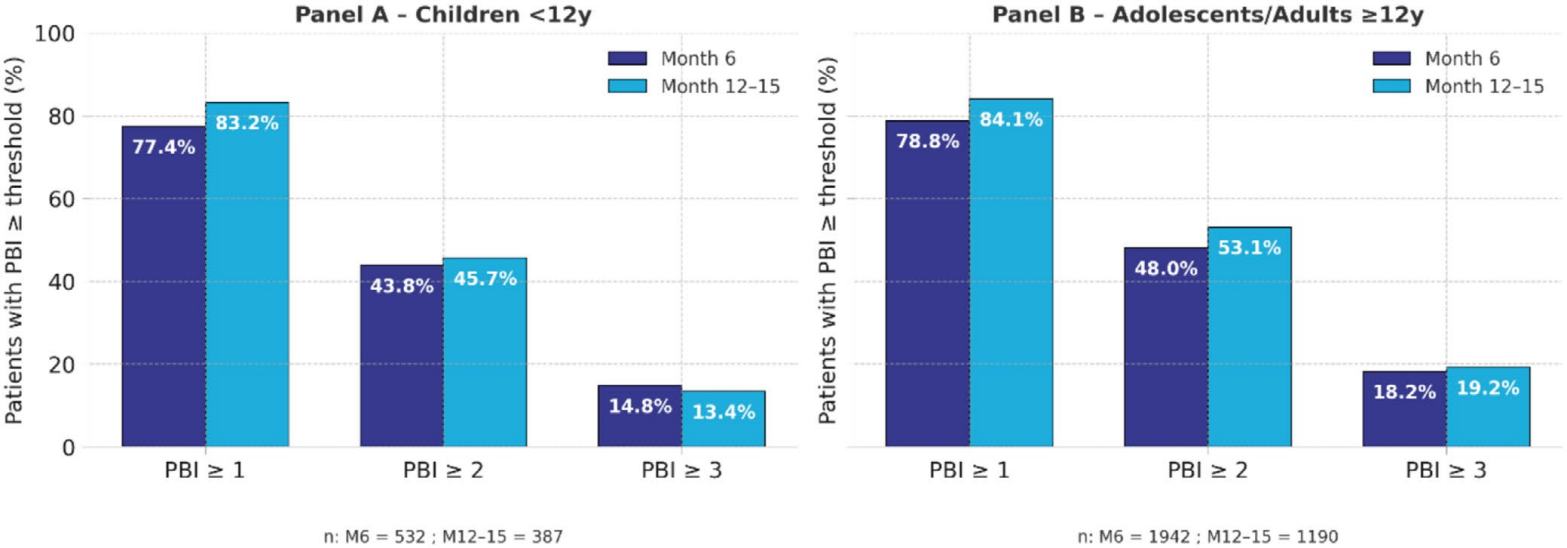
Proportion of NPP‐SLIT initiators reporting clinically meaningful and higher levels of patient‐perceived benefit (PBI) at Month 6 and Month 12–15. Panel A: Children (< 12 years). Panel B: Adolescents/adults (≥ 12 years). Bars show the percentage of initiators achieving PBI ≥ 1, PBI ≥ 2 or PBI ≥ 3 at Month 6 or at Month 12–15 by and age group. Results are displayed for two analysis populations: i) at Month 6, patients with at least two questionnaires available (baseline + Month 6); ii) at Month 12–15, patients with all three questionnaires available (baseline + Month 6 + Month 12–15).

Further descriptive analyses explored stricter thresholds of perceived benefit, including higher PBI cutoffs and analyses in patients with complete follow‐up data (Table 3) and (Figure 2). At Months 12–15, PBI ≥ 2 was achieved by 46.5% (204/439) of children and 52.7% (722/1371) of adolescents/adults, while PBI ≥ 3 was reported by 14.8% (65/439) and 19.3% (264/1371), respectively. In the subset of initiators with complete data at baseline, Month 6, and Months 12–15, results were consistent, with PBI ≥ 2 achieved by 45.7% (177/387) of children and 48.7% (579/1190) of adolescents/adults, and PBI ≥ 3 by 13.4% (52/387) and 17.4% (207/1190) (Figure 2).

**FIGURE 3:**
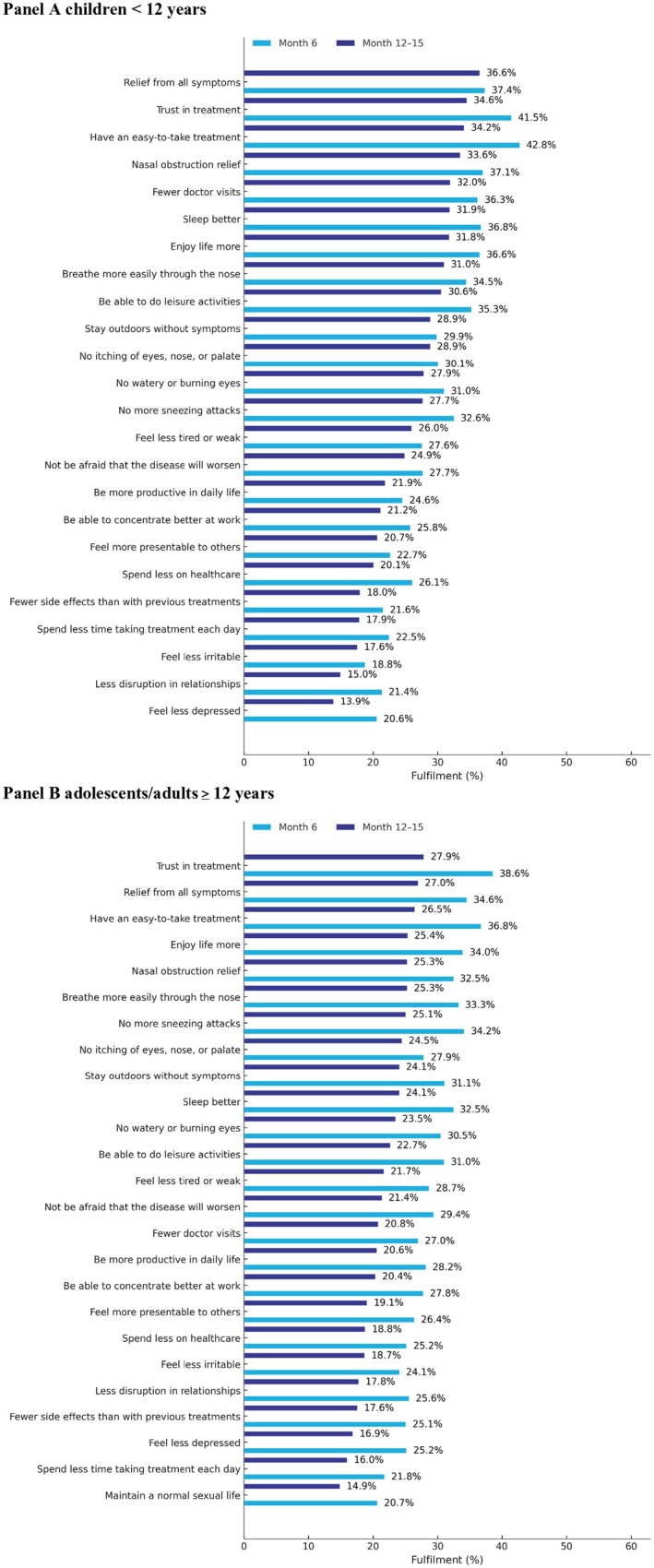
Fulfillment of all patients defined treatment benefits (PBQ items) at Month 6 and Month 12–15, by age group (initiators only). Panel A children < 12 years>. Panel B adolescents/adults ≥ 12 years. Bars show the proportion of patients who rated each expectation as “important” or “very important” at baseline (Patient Needs Questionnaire, PNQ) and subsequently reported fulfilment at follow‐up. Percentages are calculated with the PNQ item‐specific denominator. The x‐axis is truncated at 60% (no item exceeded this threshold) to enhance readability and allow clearer visual comparison across expectations. *n* (Children < 12y): Month 6 = 532; Month 12–15 = 439. *N* (Adolescents/Adults ≥ 12y): Month 6 = 1942; Month 12–15 = 1371.

Exploratory PNQ/PBQ item‐level analyses showed that the expectations most frequently reported at baseline were treatment easy to take, relief of all symptoms, trust in treatment, and nasal obstruction relief. At Month 12–15, fulfillment rates across the 25 PBQ items ranged from ~14% (e.g., improved mood, sexual life, or social aspects) to ~37% (relief of all symptoms in children and trust in treatment in adults). As the PBI is computed at the individual level as a weighted average across the items rated important at baseline, these cohort‐level item fulfillment proportions are not directly comparable with the overall PBI ≥ 1 proportion and primarily reflect heterogeneity in patients' needs and priorities. The highest fulfillment proportions were consistently observed for expectations that were also the most frequent at baseline, notably symptom relief, nasal obstruction, and sleep. Children generally reported slightly higher fulfillment rates across most domains compared with adolescents/adults (Figure 3). Subgroup analyses confirmed consistently high proportions of initiators achieving a clinically meaningful benefit (PBI ≥ 1) at Month 6 and Months 12–15 across all demographic and clinical categories. Rates were comparable across sex, asthma comorbidity, and baseline asthma control (ACT). At Month 6, the proportion of clinically meaningful benefit increased with baseline rhinitis severity (ARIA) and baseline symptom burden (T5SS) in both children and adolescents/adults. These trends remained at Months 12–15 for ARIA in children and for T5SS in adolescents/adults. Slightly higher proportions were reported in children compared with adolescents/adults.

## 4 Discussion

The ERAPP study demonstrates that patient‐perceived benefit, assessed by the PBI, was achieved by most NPP‐SLIT initiators at Months 12–15. Benefit rates remained high over time, particularly among children. This confirms the robustness of the primary finding and underlines the value of the PBI as an integrative, patient‐centered endpoint. By focusing on patients early in their SLIT course, ERAPP offers longitudinal insights into how benefit is experienced and evolves in routine practice.

A core strength of this study is the use of the PBI, which links treatment outcomes to individual expectations. Unlike conventional PROMs, the PBI captures the personal relevance and perceived value of therapy. In ERAPP, the proportion of patients reporting clinically meaningful benefit (PBI ≥ 1) remained consistently high across baseline severity levels. Item‐level analyses confirmed that the most frequently fulfilled expectations were related to nasal obstruction relief, improved sleep, and overall symptom control, particularly in children. Conversely, expectations related to fatigue, concentration, or emotional and social aspects were less frequently met, highlighting areas where complementary management strategies may be required.

PROM trajectories provided an additional perspective. Rhinitis severity (ARIA) and symptom burden (T5SS) improved over time and exceeded published MID thresholds, supporting clinically meaningful improvement. Asthma control (ACT) also improved (shift toward a higher proportion of controlled asthma among patients with evaluable ACT), whereas changes in rhinitis control (ARCT, available in patients ≥ 12 years) and daytime sleepiness (ESS, in adults) were more modest and remained below their respective MID thresholds. The partial divergence between symptom burden (T5SS) and control (ARCT) likely reflects differences in constructs and recall windows; together with robust PBI gains, this underscores the added value of the PBI as a complementary, expectation‐anchored endpoint.

These item‐level findings are in line with the PROM trajectories. The domains with the highest fulfillment (nasal obstruction relief, sleep, overall symptom relief) were consistent with PROMs showing the most pronounced or clinically meaningful changes, namely, ARIA and T5SS. Improvements in ACT and ESS were more modest and did not reach their established MIDs, although sleep‐related expectations remained among the most frequently fulfilled at the item level. Conversely, expectations related to fatigue, mood, or social functioning were less frequently fulfilled, which is expected since these dimensions are not directly targeted by AIT and are not fully captured by conventional PROMs. This coherence between PROMs and PBI strengthens the interpretability of our results and highlights how the PBI complements standard outcome measures by anchoring them to patient‐defined priorities.

Treatment satisfaction (ESPIA‐Q11) increased over time, whereas adherence (GIRERD) declined between baseline and follow‐up visits. This pattern is consistent with the well‐documented challenge of maintaining compliance in long‐term sublingual AIT [16, 17]. Although perceived benefit (PBI) remained high, one can hypothesize that sustained adherence might further amplify treatment effectiveness. Dialog between patients and physicians, regular re‐evaluation of treatment goals, and structured therapeutic education are therefore essential. In patients reporting limited benefit (e.g., low PBI), this re‐evaluation should explicitly consider whether AIT should be continued, adapted, or discontinued within a shared decision‐making process. Digital tools may also provide support: for instance, the French Drago mobile application has shown encouraging results in improving adherence and persistence in patients treated with HDM SLIT, particularly in children [18].

Exploratory analyses further illustrated the depth of benefit perceived by patients. Higher thresholds of benefit (PBI ≥ 2 and ≥ 3) confirmed that a substantial proportion of patients reported strong perceived improvements, with results consistent across patients with complete data at all timepoints. Subgroup analyses confirmed consistently high levels of benefit across sex, age group, asthma comorbidity, and baseline severity (ARIA, T5SS, and ACT). Slightly higher proportions were observed in children compared with adolescents/adults. As no formal statistical comparisons were performed, subgroup differences should be interpreted descriptively.

These findings are in line with those of prior real‐world studies in respiratory allergic diseases. In Germany, a large observational study of birch pollen SLIT including more than 1000 patients reported consistent improvements in PROMs and confirmed the relevance of assessing benefit from the patient's perspective [19]. In France, the BENEFICA study applied the PBI to evaluate the effect of antihistamines in allergic rhinitis and demonstrated that this tool was able to capture meaningful improvements in domains such as nasal obstruction, sleep, and daily functioning [7]. Together with ERAPP, these studies underscore the importance of systematically assessing patient benefit and expectations in routine allergy care. Moreover, as emphasized by Laferton et al. [20], addressing and reinforcing patients' treatment expectations is itself a determinant of therapeutic outcomes and long‐term engagement. They also echo recommendations from the French (SFA) and European (EAACI) allergy societies, which stress the need to elicit and strengthen expectations to optimize adherence and outcomes of allergen immunotherapy [19, 21]. The study has several strengths, including its very large and diverse real‐world population, the inclusion of both pediatric and adult patients, and the systematic use of validated PROMs combined with the PBI framework. Limitations include its observational design, the absence of a control group, and attrition at Months 12–15, which may introduce selection/attrition bias and overestimate benefit if more motivated or satisfied participants were more likely to remain in follow‐up. We also did not adjust for potential confounders such as seasonality (timing of follow‐up relative to pollen seasons), concomitant pharmacotherapy, disease duration, or regression to the mean; therefore, findings should be interpreted as descriptive real‐world trajectories rather than causal treatment effects. The exploratory nature of subgroups and PNQ/PBQ item‐level analyses also warrants cautious interpretation. In addition, adherence was measured with the GIRERD questionnaire, which provides a global assessment but does not capture daily fluctuations in treatment‐taking behavior.

Altogether, ERAPP highlights the relevance of the PBI as a sensitive and integrative outcome measure, capable of capturing both clinical improvement and fulfillment of individual treatment goals. While NPP‐SLIT provides robust, patient‐perceived benefits in both children and adults, optimizing long‐term adherence through education, shared decision‐making, and innovative tools such as Drago remains a key opportunity to maximize real‐world therapeutic impact.

## 5 Conclusion

In summary, the ERAPP study provides robust real‐world evidence supporting the multidimensional impact of NPP‐SLIT in patients with allergic rhinitis and/or asthma, including those with comorbid asthma. By combining subjective patient perspectives with objective disease‐control measures, ERAPP illustrates how modern AIT outcomes can, and should, be aligned with what matters most to patients. These findings reinforce the clinical relevance of the PBI as a complementary outcome alongside traditional endpoints, offering clinicians and healthcare systems a tool to personalize, monitor, and optimize AIT. As allergy care evolves toward precision and value‐based paradigms, PROMs and patient‐defined measures of success will become essential.

## Author Contributions

Davide Caimmi and Pascal Demoly were responsible for the design and scientific coordination of the study. Abdelilah Abouelfath, Régis Lassalle, Séverine Lignot‐Maleyran, Emmanuelle Bignon, Laure Carcaillon‐Bentata, and Patrick Blin were responsible for the logistics and oversaw statistical methodology and data analysis. Evangéline Clark and Julien Cottet contributed to patient recruitment and data interpretation. All authors participated in the review of the manuscript, provided input on data interpretation, and approved the final version for submission. All authors had full access to the study data and are accountable for the integrity of the results.

## Funding

This work was supported by Société Française d’Allergologie (SFA).

## Conflicts of Interest

Davide Caimmi received personal fees for advisory or speaker roles from ALK, Astra Zeneca, Stallergenes Greer, GSK, and Sanofi. Pascal Demoly reports no direct financial interests; he has received institutional grants for teaching and research activities from ALK, AstraZeneca, GlaxoSmithKline, Menarini, Puressentiel, Stallergenes Greer, ThermoFisher Scientific, and Viatris. Evangéline Clark received personal fees for advisory or speaker roles from ALK, Stallergenes Greer, and Sanofi. Julien Cottet reported direct lecturing fees from Stallergenes Greer and ALK. The remaining authors declare no conflicts of interest related to the content of this article.

## Supporting information


**Table S1:** Literature‐based MID thresholds used for interpretation of PROMs.

## Data Availability

The data that support the findings of this study are available from the corresponding author upon reasonable request.

## References

[1] M. Alvaro‐Lozano , C. A. Akdis , M. Akdis , et al., “EAACI Allergen Immunotherapy User's Guide,” Pediatric Allergy and Immunology 31, no. 25 (2020): 1–101, 10.1111/pai.13189. 32436290 PMC7317851

[2] T. Batard , C. Taillé , L. Guilleminault , et al., “Allergen Immunotherapy for the Prevention and Treatment of Asthma,” Clinical and Experimental Allergy 55, no. 1 (2025): 111–141, 10.1111/cea.14567. 39363801 PMC11791393

[3] X. Li , J. Shang , J. Liu , and Y. Zhu , “A Meta‐Analysis Investigating the Efficacy and Safety of Allergen‐Specific Immunotherapy in Respiratory Allergies,” Journal of Asthma 61, no. 11 (2024): 1337–1346, 10.1080/02770903.2024.1234567. 38687911

[4] M. A. Calderón , C. Vidal , P. del Rodríguez Río , et al., “European Survey on Adverse Systemic Reactions in Allergen Immunotherapy (EASSI): A Real‐Life Clinical Assessment,” Allergy 72, no. 3 (2017): 462–472, 10.1111/all.13050. 27718250

[5] P. del Rodríguez Río , C. Vidal , J. Just , et al., “The European Survey on Adverse Systemic Reactions in Allergen Immunotherapy (EASSI): A Paediatric Assessment,” Pediatric Allergy and Immunology 28, no. 1 (2017): 60–70, 10.1111/pai.12629. 27637414

[6] N. Franzke , I. Schäfer , K. Jost , et al., “A New Instrument for the Assessment of Patient‐Defined Benefit in the Treatment of Allergic Rhinitis,” Allergy 66, no. 5 (2011): 665–670, 10.1111/j.1398-9995.2011.02533.x. 21121931

[7] P. Demoly , M. Aubier , F. De Blay , F. Wessel , P. Clerson , and P. Maigret , “Evaluation of Patients' Expectations and Benefits in Allergic Rhinitis With the Patient Benefit Index – The BENEFICA Study,” Allergy, Asthma and Clinical Immunology 11 (2015): 8, 10.1186/s13223-015-0081-8. PMC4349226 25741366

[8] D. Caimmi , A. Abouelfath , R. Lassalle , et al., “Patient‐Perceived Benefits of Sublingual Allergen Immunotherapy: Design of the ERAPP Study,” Journal of Allergy and Hypersensitivity Diseases 5 (2025): 100033, 10.1016/j.jahd.2025.100033.

[9] J. Bousquet , N. Khaltaev , A. A. Cruz , et al., “Allergic Rhinitis and Its Impact on Asthma (ARIA) 2008 Update,” Allergy 63, no. Suppl 86 (2008): 8–160, 10.1111/j.1398-9995.2007.01620.x. 18331513

[10] R. A. Nathan , C. A. Sorkness , M. Kosinski , et al., “Development of the Asthma Control Test: A Survey for Assessing Asthma Control,” Journal of Allergy and Clinical Immunology 113, no. 1 (2004): 59–65, 10.1016/j.jaci.2003.09.008. 14713908

[11] P. Demoly , R. Jankowski , O. Chassany , Y. Bessah , and F. Allaert , “Validation of a Self‐Questionnaire for Assessing Control of Allergic Rhinitis,” Clinical and Experimental Allergy 41, no. 6 (2011): 860–868, 10.1111/j.1365-2222.2011.03685.x. 21518040

[12] P. Devillier , O. Chassany , E. Vicaut , et al., “The Minimally Important Difference in the Rhinoconjunctivitis Total Symptom Score in Grass‐Pollen‐Induced Allergic Rhinitis,” Allergy 69, no. 12 (2014): 1689–1695, 10.1111/all.12504. 25155425

[13] J. L. Justicia , V. Cardona , P. Guardia , et al., “Validation of the ESPIA Questionnaire for Patient Satisfaction With Allergen‐Specific Immunotherapy,” Journal of Allergy and Clinical Immunology 131, no. 6 (2013): 1539–1546, 10.1016/j.jaci.2013.02.005. 23352631

[14] M. W. Johns , “A New Method for Measuring Daytime Sleepiness: The Epworth Sleepiness Scale,” Sleep 14, no. 6 (1991): 540–545, 10.1093/sleep/14.6.540. 1798888

[15] X. Girerd , O. Hanon , K. Anagnostopoulos , C. Ciupek , J. J. Mourad , and S. Consoli , “Assessment of Antihypertensive Compliance Using a Self‐Administered Questionnaire: Development and Use in a Hypertension Clinic,” Presse Médicale 30, no. 21 (2001): 1044–1048. 11471275

[16] M. Park , S. Kapoor , J. Yi , N. Hura , and S. Y. Lin , “Sublingual Immunotherapy Persistence and Adherence in Real‐World Settings: A Systematic Review,” International Forum of Allergy & Rhinology 13, no. 5 (2023): 924–941, 10.1002/alr.23148. 36083179

[17] P. Demoly , G. Passalacqua , O. Pfaar , J. Sastre , and U. Wahn , “Patient Engagement and Patient Support Programs in Allergy Immunotherapy: A Call to Action for Improving Long‐Term Adherence,” Allergy, Asthma and Clinical Immunology 12 (2016): 34, 10.1186/s13223-016-0140-2. PMC4966171 27478445

[18] L. K. Tanno , P. T. V. Luong , E. Fromentin , et al., “Drago: An Innovative Mobile Application to Improve Adherence and Compliance of House Dust Mite Allergen Immunotherapy,” Journal of Allergy and Hypersensitivity Diseases 6 (2025): 100041, 10.1016/j.jahd.2025.100041.

[19] C. Blome , M. Hadler , E. Karagiannis , et al., “Relevant Patient Benefit of Sublingual Immunotherapy With Birch Pollen Extract in Allergic Rhinitis: An Open, Prospective, Non‐Interventional Study,” Advances in Therapy 37, no. 7 (2020): 2932–2945, 10.1007/s12325-020-01382-3. 32342352 PMC7467431

[20] J. A. C. Laferton , W. Rief , and M. Shedden‐Mora , “Improving Patients' Treatment Expectations,” JAMA 334, no. 2 (2025): 171–172, 10.1001/jama.2025.10041. 40465235

[21] D. Caimmi and P. Demoly , “Recommandations pour la prescription de l'immunothérapie allergénique et le suivi du patient,” Revue Française d'Allergologie 61, no. 1 (2021): 24–34, 10.1016/j.reval.2020.11.005.

