# Supplementary material for: Patient‐Perceived Benefits of Named‐Patient Product Sublingual Immunotherapy in Allergic Rhinitis and Asthma: Primary Results From the ERAPP Real‐World Cohort Study
Source: Allergy. 2026 Mar 8;81(6):2145–55. doi: 10.1111/all.70270 (PMC13256285; doi:10.1111/all.70270)
Supplement: Supplementary file 1 — Table S1: Literature‐based MID thresholds used for interpretation of PROMs. [file ALL-81-2145-s001.docx]

**Supplementary Tables**

**Table S1 –** Literature-based MID thresholds used for interpretation of PROMs

| **PROM** | **Description** | **MCID reported in literature** | **Reference** |
| --- | --- | --- | --- |
| ARIA | Allergic Rhinitis and its Impact on Asthma classification | Not applicable (categorical) | (1) |
| ACT | Asthma Control Test | 3 points | (2) |
| T5SS | Total 5 Symptom Score | ~1 point per symptom | (3) |
| ARCT | Allergic Rhinitis Control Test | 3 points | (4) |
| ESPIA Q11 | Satisfaction with care | Not standardized | (5) |
| ESS | Epworth Sleepiness Scale | 2–3 points | (6) |
| GIRERD | Treatment adherence score | Not standardized | (7) |

References: 1- Valero A, Ferrer M, Sastre J, Navarro AM, Monclus L, Marti-Guadano E, et al. A new criterion by which to discriminate between patients with moderate allergic rhinitis and patients with severe allergic rhinitis based on the Allergic Rhinitis and its Impact on Asthma severity items. *J Allergy Clin Immunol.* 2007;120:359-365. 2- Schatz M, Kosinski M, Yarlas AS, Hanlon J, Watson ME, Jhingran P. The minimally important difference of the Asthma Control Test. *J Allergy Clin Immunol.* 2009;124:719-723. 3- Devillier P, Chassany O, Vicaut E, de Beaumont O, Robin B, Dreyfus JF, et al. The minimally important difference in the rhinoconjunctivitis Total Symptom Score in grass-pollen-induced allergic rhinoconjunctivitis. *Allergy.* 2014;69:1689-1695. 4- Chiriac AM, Zhu R, Izquierdo L, Molinari N, Demoly P. The minimal important difference of the allergic rhinitis control test. *J Allergy Clin Immunol Pract.* 2022;10:1924-1926. 5- Justicia JL, Cardona V, Guardia P, Ojeda P, Olaguíbel JM, Vega JM, et al. Validation of the first treatment-specific questionnaire for the assessment of patient satisfaction with allergen-specific immunotherapy in allergic patients: The ESPIA questionnaire. *J Allergy Clin Immunol.* 2013;131:1539-1546. 6- Johns MW. A New Method for Measuring Daytime Sleepiness: The Epworth Sleepiness Scale. *Sleep.* 1991;14:540–545. 7- Girerd X, Hanon O, Anagnostopoulos K, Ciupek C, Mourad JJ, Consoli S. Assessment of antihypertensive compliance using a self-administered questionnaire: development and use in a hypertension clinic. *Presse Med.* 2001;30:1044-1048.
